# GABAergic Transmission and Chloride Equilibrium Potential Are Not Modulated by Pyruvate in the Developing Optic Tectum of *Xenopus laevis* Tadpoles

**DOI:** 10.1371/journal.pone.0034446

**Published:** 2012-04-04

**Authors:** Arseny S. Khakhalin, Carlos D. Aizenman

**Affiliations:** Department of Neuroscience, Brown University, Providence, Rhode Island, United States of America; Georgia State University, United States of America

## Abstract

In the developing mammalian brain, gamma-aminobutyric acid (GABA) is thought to play an excitatory rather than an inhibitory role due to high levels of intracellular Cl^−^ in immature neurons. This idea, however, has been questioned by recent studies which suggest that glucose-based artificial cerebrospinal fluid (ACSF) may be inadequate for experiments on immature and developing brains. These studies suggest that immature neurons may require alternative energy sources, such as lactate or pyruvate. Lack of these other energy sources is thought to result in artificially high intracellular Cl^−^ concentrations, and therefore a more depolarized GABA receptor (GABAR) reversal potential. Since glucose metabolism can vary widely among different species, it is important to test the effects of these alternative energy sources on different experimental preparations. We tested whether pyruvate affects GABAergic transmission in isolated brains of developing wild type *Xenopus* tadpoles *in vitro* by recording the responsiveness of tectal neurons to optic nerve stimulation, and by measuring currents evoked by local GABA application in a gramicidin perforated patch configuration. We found that, in contrast with previously reported results, the reversal potential for GABAR-mediated currents does not change significantly between developmental stages 45 and 49. Partial substitution of glucose by pyruvate had only minor effects on both the GABA reversal potential, and the responsiveness of tectal neurons at stages 45 and 49. Total depletion of energy sources from the ACSF did not affect neural responsiveness. We also report a strong spatial gradient in GABA reversal potential, with immature cells adjacent to the lateral and caudal proliferative zones having more positive reversal potentials. We conclude that in this experimental preparation standard glucose-based ACSF is an appropriate extracellular media for *in vitro* experiments.

## Introduction

For over two decades it has been widely accepted that in very immature forebrain neurons (before postnatal day 7 in rats, and before 10 weeks of age in humans) gamma-aminobutyric acid (GABA) has a depolarizing effect, and therefore does not necessarily act as an inhibitory transmitter as it does in the more mature brain [1]. The reason for the depolarizing action of GABA during early development can be explained by the specific expression time profile of various types of chloride cotransporters. Namely, while adult neurons express high levels of KCC2 cotransporter, NKCC1 is expressed in immature brains, resulting in higher intracellular chloride concentration, and thus a more depolarized reversal potential in immature, as compared to mature neurons [1], [2], [3]. Recent findings however, have challenged this view [4], [5], [6]. These studies showed that a relatively modest change in artificial cerebro-spinal fluid (ACSF) composition – such that it more closely reproduces *in vivo* cerebro-spinal fluid composition as described in the literature [7] – makes the effect of GABA on immature neurons more hyperpolarizing. In these studies glucose, which is used at about 10 mM concentration in standard extracellular solutions across various species, was partly substituted with different energy source molecules, such as lactate, pyruvate and the ketone body beta-hydroxybutyric acid (BHB) to account for the fact that at early stages of development blood plasma, cerebro-spinal fluid, and especially extracellular fluid immediately surrounding neural cells, contain relatively high (about 4–5 mM, if considered together) concentrations of these alternative energy sources [7], [8].

This challenge to the conventional developmental paradigm, however, has also been shown to be controversial [9], [10], [11]. Thus, the question still does not seem to be completely closed, and may eventually result in certain corrections of both theoretical framework, and common experimental practices [8]. Complicating this is the fact that different species vary in their use of various metabolites during development [7], [12], and in light of this controversy it is important to examine the developmental effects of GABA in the various animal models used to study early brain development.

The retinotectal projection of *Xenopus laevis* tadpoles is a well-known experimental preparation that is widely employed for both developmental [13], [14], [15], [16], [17], and behavioral studies [18], [19], and which undergoes rapid maturation and transformation between developmental stages 45 and 49 (addressed as s45 and s49 later in the text) [13], [14], [17]. It was reported that, similar to other experimental systems, GABA action is more depolarizing in *Xenopus* tadpoles at s45, as compared to s49 [14]. This makes it important to explicitly test whether the standard glucose-containing ACSF used in this preparation [13], [14], [20] may be having an effect on the Cl^−^ reversal potential. In this study we aim to investigate this question, first, by checking whether a change from a purely glucose-based to a mixed glucose/pyruvate ACSF has any effect on efficiency of GABAergic inhibition, and second, by testing whether these changes might be caused by differences in neuronal metabolism at early *vs*. late stages of brain development.

## Results

To estimate overall level of inhibition provided by GABAergic networks in the *Xenopus* tadpole optic tectum (OT) at different developmental stages, and for different ACSF compositions, we used two indirect albeit simple measures: the cell's overall responsiveness to stimulation (RTS), and the median inter-spike interval (ISI) of the response.

To assess RTS, we measured the maximum number of action potentials that tectal cells could generate in response to optic nerve stimulation in an isolated brain preparation. While this RTS measure is clearly an integrated one, depending on factors such as excitatory synaptic drive and intrinsic excitability of neurons [15], [21], [22], any changes in inhibitory network efficiency would also be manifested as changes in RTS [23]. Moreover, it is known from previous research that feedback regulation mechanisms tend to make RTS relatively stable across different cells within the OT, and across different developmental stages [17], thus making RTS a useful index value to probe the inhibition to excitation balance during development.

**Figure 1:**
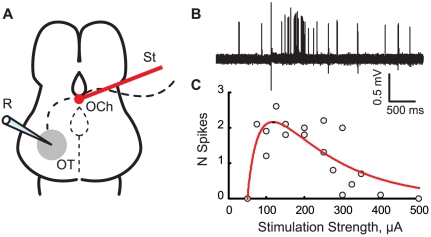
A schematic view of the preparation, and examples of the data. **A**. A simplified scheme of the preparation: R – recording electrode; OT – middle third of the Optic Tectum; OCh – Optic Chiasm; St – Stimulating Electrode. **B**. Typical responses to the optic chiasm stimulation, recorded in loose-cell-attached current-clamp mode (s49, control ACSF, 10 responses superimposed); **C**. An example of RTS calculation for one of the cells (s49, pyruvate-containing ACSF). Average spikes/stimulus values are plotted against respective stimulation strengths, and are shown together with a fit curve. Each dot corresponds to the average number of spikes/stimulus observed over 10 consecutive responses.

In our experiments, we used a whole-brain preparation and cell-attached recordings to measure the number of spikes generated by optic tectum (OT) cells in response to optic chiasm stimulation (Fig. 1A). For each cell, at least 3 different stimulation strengths were tested, and responses to at least 30 stimuli were recorded (on average 6 different stimulation strengths, and 61 stimuli respectively; Fig. 1B); the data for each cell was fit with a smooth curve (see Methods), and the maximum response (measured in spikes/stimulus) was estimated (Fig. 1C). These maximum response values were then averaged across different cells within each experimental group, and compared across different ACSF formulations and for different developmental stages.

**Figure 2:**
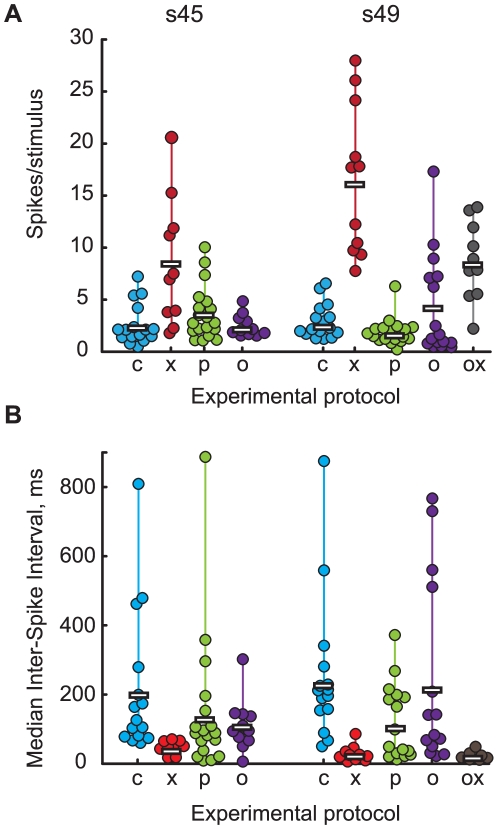
Responsiveness to stimulation and inter-spike interval values for different developmental stages and ACSF compositions. **A**. Average responsiveness to stimulation (RTS). Each colored circle represents average responsiveness-to-stimulation value for an individual cell, with cells from younger animals (s45) shown in the left group, and those from older animals (s49) – in the right group. Data obtained in ACSF of different formulations is shown in columns of different color. ACSF types are encoded in the following way (left to right): c – standard (control) ACSF; x – standard ACSF+PTX; p – pyruvate-based ACSF; o – ACSF lacking energy sources; ox – no energy sources+PTX. Horizontal bars represent mean RTS values across all cells within each data group. **B.** Median inter-stimulus intervals (ISI). Each colored circle represents median inter-stimulus interval value for an individual cell; data from animals of different age, and recorded in different ACSF formulations are given in the same order and with same labels as in panel A. Horizontal bars show average ISI values across all cells within each data group.

We first measured changes in RTS caused by the blockade of GABA_A_ receptors with Picrotoxin (PTX), as compared to control conditions. In both s45 and s49 animals elimination of GABAergic transmission lead to a significant increase in RTS: 8.5±6.2 spikes/stimulus in PTX-containing solution (n = 10) vs. 2.6±1.9 in control solution for s45 (n = 16), P = 0.003; and 16.5±7.2 spikes/stimulus in PTX-containing solution (n = 11) vs. 2.9±1.7 in control solution for s49 (n = 16), P = 2E−5 (Fig. 2A). Thus, despite the fact that GABA_A_-mediated postsynaptic currents were reported to be relatively more depolarizing in Xenopus tadpoles at earlier stages of development [14], in both age groups GABAergic networks still proved to be inhibitory. While RTS values under control conditions did not differ statistically between s45 and s49 animals (P = 0.4), RTS values observed in the PTX-disinhibited preparation were almost twice as large in s49 compared to s45 animals: 8.5±6.2 for s45 (n = 10), and 16.5±7.2 for s49 (n = 11); P = 7E−3.

We then performed a series of experiments in pyruvate-based ACSF (Fig. 2A), to test if GABAergic transmission would become more hyperpolarizing in this type of solution, increasing efficiency of inhibition, and reducing RTS values. No significant difference in RTS was observed in pyruvate-based ACSF *vs.* control experiments in s45 animals: 3.9±2.6 spike/stimulus in pyruvate-based ACSF (n = 19), 2.6±1.9 in control (n = 16, P = 0.1); however in older s49 animals pyruvate ACSF did slightly reduce RTS in comparison with controls to 1.9±1.3 spike/stimulus in pyruvate-based ACSF (n = 18) *vs.* 2.9±1.7 in control (n = 16; P = 0.04). For the pyruvate-based ACSF RTS observed in s49 animals was also significantly lower than that in s45 animals (P = 2e−5).

To check if pyruvate-driven decrease in RTS for s49 was caused by a better availability of energy sources in enhanced glucose+pyruvate ACSF, or by some other factors, we performed a series of recordings in ACSF that lacked any energy sources (no glucose, no pyruvate; Fig. 2A). We found that RTS values in energy-deprived preparations were not statistically different from controls for both s45 (2.5±1.1, n = 11, P = 0.6) and s49 (4.2±4.9, n = 16, P = 0.5). Despite complete unavailability of energy sources from modified ACSF, preparations stably responded to stimulation for about same amount of time as in control experiments (3–4 hours), demonstrating no significant rundown of responses with time (RTS values calculated separately during first and second halves of each experiment were not statistically different, P>0.1 for both Mann–Whitney U test, and Wilcoxon signed rank test for the direction of change). Elimination of energy sources did however increase RTS variability for s49 (P<0.001, Two-sample F-test for the equality of variances), but not for s45 experiments.

Finally, to ensure that inhibitory circuits in our preparation were not compromised too severely by energy source deprivation, and also to assess maximum RTS that could be practically achieved in absence of glucose and pyruvate in ACSF, we performed a series of recordings in energy source-free, but PTX-containing external solution (no glucose, no pyruvate, +PTX) in brains from s49 animals (Fig. 2A). Average RTS under this experimental condition (8.7±3.8, n = 10) was statistically higher than in both control (n = 16, P = 4E−4) and energy source-deprived ACSF (n = 16, P = 0.01), but was statistically lower than in glucose and PTX-containing ACSF (n = 11, P = 3E−4), proving that although energy sources unavailability had some moderate effect on the neurons, still the GABAergic inhibition was not very sensitive to the energy deprivation, and the network functioned well below the maximal possible level of activation.

As an alternative way to assess the overall level of inhibition in the OT for different experimental conditions we compared median inter-spike interval (ISI) values for responses of OT cells to optic chiasm stimulation. The distribution of ISIs is known to be a good indicator of burst-like spiking patterns (also known as barrages, or neuronal avalanches) in neural networks, with an increase in time-locked bursting leading to a decrease in the median ISI [24]. On the other hand, the relative contribution of burst-like patterns to network activity is known to depend on the excitation/inhibition balance [25], with bursts becoming more prevalent as inhibition is decreased, and long time before large scale seizure-like correlated spiking could be detected [26]. Conforming with these general considerations, the blockade of inhibition in OT of developing *X. laevis* is known to make OT responses more burst-like [17], making median ISI an attractive OT inhibition level indicator.

In line with changes in RTS, the ISI of OT cells was significantly lower in PTX-containing ACSF (47±16 ms, n = 10 for s45; 30±20 ms, n = 11 for s49) than in control ACSF (210±206 ms, n = 16 for s45; 244±209 ms, n = 16 for s49) for both s45 (P = 7e−5) and s49 (P = 3e−5) tadpoles (Fig. 2B). In PTX-containing ACSF the ISI values observed in s49 animals were also significantly lower than that for s45 animals (P = 0.045). ISI of responses recorded in energy source-free and PTX-containing external solution in s49 animals (22±10 ms, n = 10), were significantly lower than that recorded in control conditions (P = 2e−6). Conversely, the values of ISI recorded in energy-free ACSF (112±76 ms, n = 11 for s45; 230±269 ms, n = 15 for s49) were not significantly different than the control values for respective developmental stages (P>0.1), and no significant differences were observed between s45 and s49 developmental stages for both control and energy-source-free solutions (P>0.4). To sum up, for all comparisons performed in control, PTX-containing, energy-free, and energy-free+PTX ACSF solutions the ISI values were significantly different when and only when the RTS values were also significantly different, providing a convincing cross-validation of the results.

ISI of responses recorded in pyruvate-contaning ACSF was not different from that recorded in control ACSF for s45 (142±204, n = 19, P = 0.094), but there was a statistically significant difference between ISI of responses recorded in control and pyruvate-containing ACSF for s49 (121±109, n = 16, P = 0.023). The direction of this change in ISI was unexpected however: while for PTX-containing formulations of ACSF increases in RTS were accompanied by decreases in ISI, for pyruvate-containing ACSF at s49 both RTS and ISI values did decrease. Thus, the s49 OT cells in pyruvate-containing ACSF were observed to become less spiky than in control ACSF, but at the same time seemingly more prone to burst-like activity.

In the mammalian cortex, it has been suggested that inhibition may also serve to improve the precision of synaptically-evoked spiking through activation of feedforward inhibitory networks [23]. Hence another possible way to assess the effect of inhibition would be to measure the latency of the response onset (the median latency of the first spike in the evoked spike train) and the half-length of the response, defined as a difference between the median latency of the response and the median latency of the first spike in the train. We compared first spike latencies, recorded in ACSF of different composition from animals at different developmental stages, and found, that ACSF composition did not affect this value significantly (data not shown), which was likely due to the fact that the initial spike latencies were very variable across cells, making any meaningful comparisons difficult. No differences in train half-length was found between the recordings from control, PTX-containing, or energy-free ACSF solutions, as well as for control ACSF vs pyruvate-containing ACSF for s45. For s49 however the spike trains recorded in pyruvate-containing ACSF were significantly shorter (with half-length of 40±76 ms) than that recorded in control ACSF (91±74 ms; P = 0.01).

**Figure 3:**
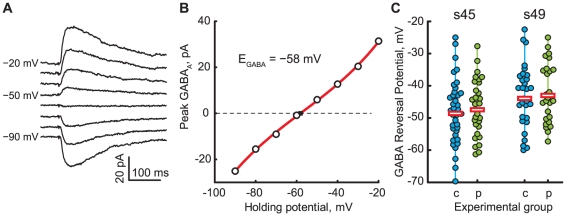
Measurement of GABAR reversal potential. **A.** Example of currents evoked by local GABA application at different command potentials (stage 45, control ACSF, central third of OT). **B.** IV-curve of GABA-evoked currents from panel A, with amplitudes shown against the command potential, polynomial fit of these amplitudes, and an estimation for GABAR reversal potential E_GABA_. **C.** E_GABA_ values observed in all cells recorded (n = 122), shown separately for stage 45 (left) and stage 49 (right) animals; control ACSF (“c”, blue) and pyruvate-containing ACSF (“p”, green). Horizontal bars show average E_GABA_ values across all cells in a data group.

In contrast to previous findings [14], we found that the action of GABA appeared to be inhibitory in the younger s45 tadpoles, and since the effect of pyruvate on GABAergic transmission was not clearly evident from our cell-attached recordings, we decided to attempt replicating results from [14], by applying GABA locally with a micropipette, and recording GABA-evoked currents at different command potentials in a gramicidin-perforated patch configuration [27]. The gramicidin patch configuration allows us to electrically access the inside of the cell, without altering the native Cl- concentration. We estimated the GABAR reversal potential (E_GABA_) from I–V curves generated by recording GABA-evoked currents at various membrane potentials (Fig. 3A,B). We found that the E_GABA_ was similar at s45 than at s49 for cells recorded in control glucose-containing solution (Fig. 3C): average E_GABA_ = −47.8±10.0 mV at s45 (n = 37) vs. −43.2±9.7 mV at s49 (n = 30); P = 0.06. Substitution of 5 mM glucose in ACSF by 5 mM pyruvate did not affect E_GABA_ at either stages (Fig. 3C): in pyruvate-containing ACSF average E_GABA_ = −46.7±8.9 mV at s45 (n = 29; P = 0.6 vs control), and −42.3±9.2 mV at s49 (n = 26; P = 0.7 vs control).

To verify these results, we used an alternative approach, estimating E_GABA_ by detecting and measuring amplitudes of spontaneous inhibitory postsynaptic currents (sIPSCs), recorded in OT cells through a gramicidin perforated patch at different command potentials. The results of these measurements were consistent with those obtained through GABA application: E_GABA_ estimations observed in control solution at different stages of development did not differ significantly over development (−40.8±9.4, n = 24 at s45; −45.1±10.8, n = 23 at s49; P = 0.1). E_GABA_ also did not differ between control and pyruvate-containing ACSFs at both stages (E_GABA_ in pyruvate-containing ACSF = −42.4±9.8 at s45, n = 16; P = 0.1 vs. control; and −46.0±10.9 at s49, n = 11; P = 0.5 vs. control). In a subset of cells where both application-induced and miniature spontaneous GABA currents were measured, both types of E_GABA_ estimations correlated significantly (r = 0.42, P = 8e−4, n = 60), further supporting the validity of our results.

**Figure 4:**
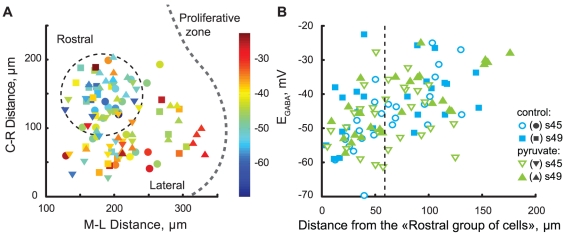
Spatial gradient of GABAR reversal potential in the OT. **A.** All cells that had their position within OT recorded (n = 105), for both stages and ACSF formulations, shown projected onto open right OT outline. Rostral direction is up, caudal is down, medial is left, lateral is right. Circles stand for cells recorded in control ACSF at stage s45; squares – control ACSF s49; down triangles – pyruvate-containing ACSF s45; up triangles – pyruvate-containing ACSF s49. Color of the marker encodes E_GABA_ measured in each of the cells, with blue corresponding to more negative, and red – to more positive values (see color-bar on the right). Subset of cells further referred to as the “Rostral group” is encircled with a dashed circle. **B.** E_GABA_ observed in OT cells as a function of distance from the center of the “Rostral group” shown on the left. Blue for control ACSF; green for pyruvate-containing ACSF; marker shapes follow same conventions as in the left panel. The threshold distance limiting the “Rostral group” is shown as a dashed line.

**Figure 5:**
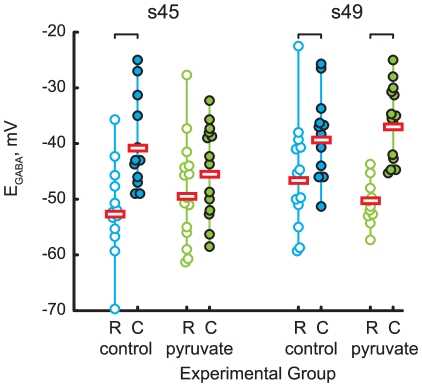
Comparison of GABAR reversal potentials in cells grouped by their location in the OT. Stage 45 cells are shown on the left; stage 49 – on the right. C – caudal group of cells (adjacent to the proliferative zone), R – rostral group of cells. Blue – control ACSF, green – pyruvate-containing ACSF. Statistically significant differences are marked with a square brake on top. Horizontal bars represent average E_GABA_ values across all cells in a data group.

For 105 out of 122 cells, in which E_GABA_ was measured through GABA application, we also registered their location within the OT, allowing us to look at possible spatial patterns in E_GABA_ distribution. Overall, we found that cells with more depolarized values of E_GABA_ tended to be located near the OT proliferative zone (laterally and caudally) where tectal cells are more immature [13], while cells with most negative E_GABA_ values were found in the rostral-medial part of the OT (Fig. 4A). To quantify this distribution, we defined a region within the rostral-medial population of the OT cells which was furthest from the proliferative zone (180 µm laterally from the midline, and 150 µm rostrally from the rostral side of the caudal proliferative zone lip), and calculated distances from this point to each OT cell we studied. E_GABA_ values clearly became more depolarized as this distance increased (Fig. 4B). For the sake of further statistical analysis, a threshold distance of 60 µm was chosen to split the population of OT cells into “rostral” (mature) and “caudal” (immature) subpopulations. The difference in E_GABA_ between these populations (Fig. 5) was found to be significant for s45 cells in control ACSF (−51.8±8.3, n = 13 for rostral, −40.0±8.4, n = 12 for caudal group, P = 2e−3), s49 in control ACSF (−45.8±10.0, n = 13 for rostral, −38.5±7.7, n = 12 for caudal group, P = 0.04), and s49 in pyruvate-containing ACSF (−49.4±5.8, n = 12 for rostral, −36.1±6.8, n = 14 for peripheral group, P = 2e−4), but not for s45 cells in pyruvate-containing ACSF (−48.6±9.2, n = 15 for rostral, −44.7±8.3, n = 14 for peripheral group, P = 0.19). However no difference was found between E_GABA_ values measured in standard ACSF at different stages, but in equivalent locations, nor for values obtained from equivalent locations and same developmental stage, but in different ACSFs.

## Discussion

This study presents three novel findings. First, we compared the role of GABAergic transmission in limiting synaptically evoked spiking activity in stage 45 and 49 tadpoles. We found that at both stages GABA transmission was significantly inhibitory, and that overall, the reversal potential for GABA currents did not differ significantly across developmental stages, despite previous reports indicating that the reversal potential for GABAR-mediated currents is more depolarizing in younger animals [14]. At the same time, the amount of disinhibition was found to be greater in older tadpoles.

Our second finding addresses the role of energy-substrate availability in determining the internal chloride concentration, and thus the inhibitory role of GABA transmission, during early development. We found that introduction of pyruvate into ACSF does not significantly change average GABA reversal potential in OT, and does not affect spiking output of tectal neurons at stage 45. We also showed that the spiking output of OT neurons is preserved in preparations completely deprived of energy sources. It suggests that the problem of inadequate energy availability in glucose-based ACSF, as it has been suggested for other systems [4], [5], [7], is not relevant for our preparation during the experimental time frame studied (4–6 h). However we did see a small but significant decrease in spike output, complemented by a switch to a more temporally localized response patterns, in pyruvate-supplemented media during s49.

Finally, we have shown that GABA reversal potential in OT cells follows a distinct spatial distribution, with more hyperpolarized E_GABA_ values found in rostromedial cells, and more depolarized E_GABA_ values observed in cells located near the OT proliferative zone (both laterally and caudally). This spatial distribution of E_GABA_ was preserved across development, and was largely not affected by changes in ACSF composition. The distribution of E_GABA_ observed in our experiments conforms with a known sequence of OT cell maturation in which cells are born at the proliferative zone, and become more mature towards the rostral-medial region of the OT [28], [29].

Comparison of RTS and ISI values for animals of different age in control and PTX-containing ACSF showed, that although with GABA transmission blocked neurons at s49 generated on average 2 times more action potentials in response to stimulation than at s45, and did so in bursts of significantly higher frequency, under normal conditions neither responsiveness nor ISI did not differ across these stages. One possible interpretation of these data may be that GABAergic networks become more efficient with age, serving as a homeostatic mechanism to maintain the average output of tectal networks flat across these developmental stages. Previously we had shown that a marked increase in excitatory drive to tectal neurons, accompanied by a complimentary decrease in intrinsic excitability, takes place between stages 45 and 49 [17]. This change of network properties results in stable input-output functions, and suggests a homeostatic mechanism to regulate neuronal throughput. Our current findings show however that, at least under these present experimental conditions, this mechanism may be insufficient to regulate neuronal output, since in experiments with PTX-containing ACSF s49 neurons still fired more spikes in response to optimal stimulation than younger ones. It may be therefore beneficial for the OT system to have several layers of homeostatic regulation, mediated by both individual OT cells, and surrounding inhibitory networks. Our data also suggest that the larger amount of disinhibition, as observed in older tadpoles, is not due to changes in GABAR reversal potential, but may be mediated by other mechanisms, such as improved shunting of postsynaptic currents due to developmental changes in electrotonic interaction between GABAR and action generation area [30].

The reduction of RTS in pyruvate-based solution, which was observed in preparations from s49 animals, was formally in agreement with recent findings from Zilberter and coworkers [4], [5], but could not be explained by the mechanism proposed by these authors, as the reversal potential of GABAR-mediated currents did not become more negative when the preparation was immersed in ACSF containing physiological concentrations of pyruvate. The reduction of RTS in our experiments was most probably mediated by factors unrelated to GABAergic transmission, such as modulation of neuronal excitability by pH_in_ [9], [31]; weak-acids mediated modulation of glutamate release [32]; effects of pH_in_ on the high-frequency burst firing [33], [34], or by any combination of these effects.

In the same vein, the decrease in median ISI of s49 OT responses that was observed in pyruvate-based ACSF, as compared to control ACSF, cannot be attributed to strengthening in GABAergic inhibitory networks, as GABA reversal potential was not affected by ACSF composition at this developmental stage. This change may be explained, however, by effects of pyruvate onto neuronal excitability [9], [31], [33], [34] and/or probability and dynamics of glutamate release [32], which could easily reduce or eliminate long-latency polysynaptic spiking at a network level.

To further strengthen the case against potential importance of pyruvate for the energy homeostasis in our preparation (unlike preparations of mammalian brains [5], [7]), we have directly demonstrated in experiments with glucose-free ACSF that unavailability of energy sources did not make tectal neurons more excitable on average. Moreover, much to our surprise, the preparation proved to be extremely tolerant to energy source deprivation, and neither became overexcitable, nor stopped responding to stimulation under these harsh experimental conditions, with increase of response variability across neurons being the only consequence of the drastic energy pool reduction. This relative insensitivity of immature Xenopus brains to energy availability is probably related to the low metabolism level generally observed in these ectothermic animals [35].

The comparison of results obtained in preparations without available energy sources to responses recorded in “energy-source deprived+PTX” solution proved that inhibition was still intact when neither glucose nor weak acids were available, and PTX was able to considerably increase RTS, and decrease ISI in OT cells.

In contrast to our previous research [36], neither response half-length, nor median latency of the response was different for s45 and s49 tadpoles in PTX-containing ACSF. The reason for this disagreement however is that in [36] the strength of optic chiasm stimulation was fixed at the level that maximized postsynaptic current-related component of the field potential [17], while in our experiments the responses analyzed for their length and latency were acquired at a wide range of stimulations strengths. For the same reason the RTS values reported here are higher than those from [36] for both s45 and s49 (e.g. 16 spikes/stimulus for s49 tadpoles in PTX-containing ACSF in our experiments vs. 5 spikes/stimulus in [36]), and we show a significant increase in RTS for PTX-containing ACSF between s45 and s49 experiments, while no change was found in [36]. This variation of results demonstrates that the maxima in RTS for each individual neuron at one hand, and in postsynaptic input for the network as a whole at the other hand, may occur at different levels of visual input activation. It is also important to mention that our methodology of RTS assessment may have favored effects of excitation over that of inhibition (as RTS was measured at the peak of each neuron's response curve, Fig. 1C). It may be that actual activation of OT cells by retinal inputs, as it happens in vivo, operates either on the left (weaker) or right (stronger activation) shoulders of the RTS curve, where interplay between excitation and inhibition could result in developmental patterns slightly different from those described in this study.

The fact that in our study we have not observed changes in E_GABA_ with development stands in apparent contrast with previous reports [14]. One possible reason for this discrepancy may be in subtle differences of spatial sampling of OT cells in our studies. We now show that GABAR reversal potentials observed in individual OT cells strongly depend on the location of these cells within the OT, and although it has long become a standard practice to restrict the recordings to middle half [14], or middle third of the tectum [17], [36], this “middle third” seems to be quite heterogeneous in terms of GABA action (Fig. 3A), which can potentially introduce bias into measurements.

Another possible way to explain the discrepancy between our observations of E_GABA_ and those published previously is to consider that different strains of *X. Laevis* used in these studies (wild type animals in this current study; albinos in [14]) may follow different maturational profiles. Albino tadpoles are known to have poorer vision than the wild type animals, presumably due to reduced pigmentation in their eyes, which is manifested in them being unable to demonstrate some kinds of visually-mediated behaviors, routinely observed in wild-type animals (C. Aizenman, unpublished observations). Absence of eye pigmentation could thus mean consistently increased spatial and temporal uniformity of inputs to the OT, which would further contribute to differences in OT circuitry maturation.

In any case, the findings presented in this publication agree with previous reports in the most crucial point: we report E_GABA_ to be changing from more positive and depolarizing to more negative and hyperpolarizing values as neurons mature. We have observed this change as a spatial gradient co-aligned with the developmental gradient in the OT [13], while previous studies might have described this same effect in terms of changes in E_GABA_, as measured in the same relative position within the OT at different stages of development [14].

To sum up, the results of this study show that partial substitution of glucose by pyruvate in ACSF used for recordings from s45-s49 *Xenopus* tadpoles does not improve energy availability in the brain, and does not largely affect the balance of excitation and inhibition. Thus it does not seem to be necessary to make any changes to current standard experimental protocols and ACSF formulae for this preparation, regardless of what consensus is reached on the mechanisms of lactate and pyruvate action on immature neural circuits in mammalian systems. We also call for caution in assuming a distinct profile of GABAR currents maturation in *Xenopus* tadpoles tectum, as this seems to be strongly dependent on the area of the tectum where the recording is performed, as well as, potentially, the strain of animals used.

## Materials and Methods

### Preparation

All animal experiments were done in accordance with Institutional Animal Care and Use Committee standards. Wild-type *X. laevis* tadpoles were raised on a 12 h light/dark cycle at 23°C in 10% Steinberg's solution. Developmental stages were determined according to Nieuwkoop and Faber [37]; tadpole brains were prepared as described in [15]. In brief, tadpoles were anesthetized in 0.01% tricaine methane sulfonate (MS-222). To access the ventral surface of the tectum, brains were filleted along the dorsal midline and dissected in HEPES-buffered extracellular solution. Composition of external solutions (in mM), for control series of experiments: 115 NaCl, 4 KCl, 3 Cacl2, 3 MgCl2, 5 HEPES, 10 glucose; for experiments with pyruvate instead of 10 glucose we used 5 glucose+5 pyruvate; for experiments with no energy sources glucose was removed, and no substitutes were added; in all cases we brought pH to 7.2, and osmolarity to 250 mOsm. For all series of experiments except that with inhibition block, dissection and recording were performed in the same solution; for experiments with inhibition block, in order to prevent possible brain rundown, 0.1 mM PTX was added to base solution 15 minutes before the recording. Brains were pinned to a submerged block of Sylgard in a recording chamber and maintained at room temperature (24°C). To access the tectal cells, the ventricular membrane surrounding the tectum was carefully removed under the microscope using a broken glass pipette.

### Electrophysiology

A bipolar stimulating electrode (FHC, Bowdoin, ME) was placed on the optic chiasm to activate RGC axons. Stimuli of 0.1 to 1 mA and duration of 0.2 ms were provided every 20 s. Cells were visualized using a Nikon (Tokyo, Japan) Eclipse FN1 light microscope with a 60× water-immersion objective, in combination with a Infinova (Monmouth Junction, NJ) CCD camera. Tight or loose cell-attached current-clamp recordings were made using glass micropipettes (9–13 MΩ) filled with external solution. We avoided patching cells that looked bigger and rounder than their neighbors, to ensure that no mesencephalic trigeminal neurons, but only the principal tectal neurons are included in the study [21]. To compare tectal neurons across the different stages of development, we restricted our recordings to the middle third of the tectum, thus avoiding developmental variability along the rostro-caudal axis [13]. To make data from different experiments comparable, all preparations were used for approximately same time of 3–4 hours. Cells that did not respond reliably to stimulation, even if they were visually healthy, formed cell-attached clamp of normal resistance, and spiked in response to membrane stretching, were not included into statistics (i.e. such cells were not counted as cells with zero spikes/stimulus). Occurrence of such non-responsive cells was about the same for all recording protocols (30–40% of total cells approached), and so should not affect our conclusions. Drugs and chemicals were obtained from Sigma (St. Louis, MO).

### Data processing and analysis

Electrical signals were measured with a Digidata 1440A and a Multiclamp 700B amplifier (Molecular Devices, Union City, CA), digitized at 10 kHz, and acquired using pClamp 10 software. In total 127 cells from 32 animals were recorded; on average 61 sweeps were recorded from each cell (from 30 to 350 for individual cells). Data was later processed offline, in a set of custom Matlab routines and functions. For analysis purposes, all animals with developmental stages ranging from 43 to 46 were pooled together, and addressed as s45, while all animals at stages s48 and s49 were pooled and addressed as s49. To ensure validity of comparisons across experimental groups, average animal stage was traced for each group, and no experimental group across different s45 or s49 experiments was statistically younger or older than the others. Data was filtered with a band-pass filter (a combination of median filter and Savitzky-Golay filter, with a pass band from about 100 to 900 Hz), to remove high-frequency noise, and low-frequency components such as stimulation artifact, changes of cell membrane potential for tight cell-attached recordings, and field potentials for loose cell-attached recordings. Spikes were identified using a simple threshold algorithm, where threshold was set manually at about 1/3 of a typical spike height. To assess “Maximum possible response” (RTS) of each cell, average spike/stimulus values, obtained for different stimulation strengths, were fit with a smooth curve. Depending on what type of curve provided a better fit, either a “saturation curve” y = c−a/(x−b), or an “optimal stimulation strength curve” y = [c−a/(x−b)]·exp[−(x−b)/d] (Fig. 1C) was used (where x stands for stimulation strength, while a, b, c and d are fit parameters). RTS was then estimated as either horizontal asymptote of this curve (in case of the “saturation curve” fit), or its maximum (in case of the “optimal stimulation” fit). For median ISI comparisons only cells with more than 5 observed ISIs were considered (3 out of 127 total cells did not satisfy this criterion, and were excluded from the set for ISI analysis).

### Gramicidin recordings

The procedures were based on those described in [14], with some modifications. We used a standard internal solution, in mM: 100 potassium gluconate, 8 KCl, 5 NaCl, 1.5 MgCl2, 20 HEPES, 10 EGTA, 2 ATP and 0.3 GTP, pH 7.2, 250 mOsm (expected observed E_Cl−_ of −66 mV, assuming junction potential of +12 mV). Gramicidin (Sigma-Aldrich, a mixture of gramicidins A, B, C, and D) was dissolved in dimethylsulfoxide to produce a stock solution of 5 mg/ml. Before the experiment, 2 µl of this stock solution was added to 0.5 ml of pre-filtered intracellular solution, and sonicated for 2 min to produce a final gramidicin concentration of 20 µg/ml; the solution was then kept on ice till the end of experiment (for 3–5 h). To pharmacologically isolate GABA currents, N-methyl-D-aspartate (NMDA) and α-amino-3-hydroxy-5-methyl-4-isoxazolepropionic acid (AMPA) receptor blockers D-2-amino-5-phosphonovaleric acid (D-APV, 50 µM) and 2,3-dihydroxy-6-nitro-7-sulfamoylbenzo[f]quinoxaline-2,3-dione (NBQX, 20 µM) were added to the external solution. To prevent recording micropipettes (9–13 MΩ) from being clogged with precipitating gramicidin, they were front-filled by brief (10–20 s) immersion of tips into standard filtered internal solution (front-filling happened through the action of capillary forces), and then back-filled with gramicidin-containing solution. To prevent gramicidin spillover, no positive pressure was applied when the tip was brought to the cell. After successful formation of a tight membrane seal, gramicidin perforation was evaluated by monitoring the parameters of a test step (through estimation of access resistance R_a_, membrane resistance, R_m_, and membrane capacitance, C_p_), as well as the amplitude of responses to GABA application. First recordings were usually possible in about 5–10 min after the seal formation (with mean test step parameters of R_a_ = 51±25 MΩ, R_m_ = 3.9±2.6 MΩ, C_p_ = 7±3 pF), and were continued for as long as 30 min (mean test step parameters by the end of protocol R_a_ = 62±25 MΩ, R_m_ = 2.2±1.3 MΩ, C_p_ = 12±3 pF). E_GABA_ was calculated from amplitudes of GABA-evoked currents (averaged over a 50 ms window around the peak) recorded at different potentials (from −90 to −20 mV, with step of +10 mV), as an intersection between the x-axis and the 3 d order polynomial fit of the observed amplitudes. Potentials were not adjusted for junction potential (expected value of +12 mV for our set of solutions). GABA (100 µM, Sigma) was applied every 30 s through a micropipette (same tip size as for patch micropipettes) connected to a picospritzer (Toohey Company, Fairfield, NJ; 30 psi for 3 ms). The position of micropipette tip relative to the cell was adjusted so that the amplitudes of evoked currents at −90 mV were in the range of 20–100 pA (average of 54±46 pA). Total of 122 cells (22 animals) were recorded. For each cell E_GABA_ measurement protocol was repeated 2–3 times (average of 2.8). For all recordings, we also attempted to detect miniature postsynaptic GABA-currents using a custom Matlab routine (based on [38]), and to use amplitudes of these miniature events as an alternative way of E_GABA_ estimation, subjecting them to the same polynomial fit algorithm as described above for evoked currents. As amplitudes of miniature GABA currents are much lower than those of evoked currents, reliable E_GABA_ estimation based on these measurements was possible only in a subset of recordings (60 out of 122 cells), and only for protocols recorded 15–20 minutes after patching the cell. In an additional set of early experiments (14 cells, 5 animals) only miniature currents were recorded (GABA was not applied); the results of these recordings were pooled with the main set of miniature currents measurements where possible. In the majority of cells in the main sequence of experiments (105 out of 122) their position within the OT was recorded, and the cells were sampled broadly (not only from the middle of the tectum), while 17 cells were recorded without precise indication of their position, but from the middle third of the tectum. For quantification purposes, the position along the medial-lateral axis was measured from the brain midline, and the position along the caudo-rostral axis was measured from the rostral edge of the OT caudal proliferative zone lip (Fig. 4A), as this morphological feature seemed to be most consistent across developmental stages, as well as most spared in individual preparations.

The following criteria were used to ensure the integrity of the perforated patch, and validity of reported E_GABA_ measurements: 1) After measurement of E_GABA_, brief suction was applied to switch from perforated to the whole-cell configuration, and a change in access parameters was observed (an increase in Cp and a decrease in Ra, to mean values of R_a_ = 26±10 MΩ, R_m_ = 2.1±1.6 MΩ, C_p_ = 16±4 pF). Cells, in which this change in access parameters was not found, were discarded. 2) Cells that achieved access values of Cp>10 pF and Ra<40 MΩ within first 5 min after patch were considered “spontaneous whole cell”, and were discarded. We also relied on the following considerations as an indirect support for the validity of E_GABA_ potentials observed at s45 in this study (as compared to more negative values reported previously [14]): 3) In 35 out of 40 cells, in which E_GABA_ was measured in both perforated and whole-cell configurations, E_GABA_ measured in whole-cell mode was more positive than in the perforated patch mode. Thus, had even the spontaneous breakthrough occurred, it would shift the estimation of E_GABA_ up, and not down, while the E_GABA_ values we observed were more negative than those previously reported for same stages of development. 4) In cells, deliberately brought into whole-cell configuration at the end of recording protocol, E_GABA_ estimation measured in whole-cell mode did not correlate with E_GABA_ measured in the perforated patch mode, and was not more positive near the proliferative zone, as opposed to the rostromedial part of the OT. This observation indicates in favor of differences in E_GABA_ estimation in perforated patch mode being a reflection of underlying differences in “target” E_GABA_ values, and not of varying abilities of different OT cells to withstand alterations in [Cl^−^]_in_ during the experiment (respectively either washout of Cl^−^ through the whole-cell patch, or changes in [Cl^−^]_in_ as a result of GABA application in the perforated patch configuration). 5) Cells, deliberately brought into whole-cell configuration with a pipette filled with gramicidin-containing solution, deteriorated quickly, and usually looked “dead” (spherical in shape, with granulated, highly contrasted interior) in about 5 minutes after the breakthrough, presumably because of the massive perforation of cell membrane with gramicidin from inside. This change in appearance did not occur spontaneously in cells used in this study, which indicates in favor of seal integrity. 6) In a separate set of experiments (n = 6; data not shown) we checked the typical rate of Cl^−^ washout, and related E_GABA_ change, after cells were patched in a whole-cell configuration with a pipette filled with low-chloride, no gramicidin internal solution ([Cl^−^]_in_ of 1 mM). The command potential was changed every 3 s in a cycle of −80, −60 and −40 mV; amplitudes of miniature postsynaptic GABA currents were measured, and their change in time for each command potential was fit with an exponential decay curve, thus allowing a continuous estimation of the chloride reversal potential. In these experiments E_GABA_ demonstrated 80% of drop to its new stable value within first 1–2 min after the breakthrough, making it highly unlikely that a spontaneous breakthrough in any of the cells in the main set of experiments would remained unnoticed.

### Statistical methods and data presentation

All types of data (RTS, ISI, median 1st spike latency, E_GABA_ etc.) were calculated at cell level; data points for individual cells were then compared across groups. Unless specified otherwise, for all comparisons we used a non-parametric Mann–Whitney U-test. We preferred a non-parametric test to a traditional Student's T-test, as distributions of values in experimental groups did not seem to be approaching natural distribution, but looked as asymmetric distributions with outliers, suggesting use of a non-parametric test. All statistical analysis was performed in MathWorks Matlab with Statistics Toolbox (version 7.3 R2010a). P-values are given together with number of cells for both groups compared, and P-value of 0.05 is used as a significance threshold. As size of data sets for different values and experimental conditions is different, all average values are given together with their STD, not SEM.
